# Prevalence of Child Functional Difficulties and Its Associated Factors in Bangladesh: An Application of Count Regression Model

**DOI:** 10.1155/2022/6328522

**Published:** 2022-12-27

**Authors:** Md. Jakaria Habib, Md. Ismail Hossain, Iqramul Haq, Md. Injamul Haq Methun, Md. Saifullah Sakib

**Affiliations:** ^1^Department of Statistics, Jagannath University, Dhaka 1100, Bangladesh; ^2^Department of Agricultural Statistics, Sher-e-Bangla Agricultural University, Dhaka 1207, Bangladesh; ^3^Statistics Discipline, Tejgaon College, Dhaka 1215, Bangladesh

## Abstract

Children that are mentally and physically healthy have a higher quality of life and are better able to function in their daily lives. Therefore, this study is aimed at investigating associated factors causing functional difficulties in male and female children ages 5-17 years. This study used data from a nationally representative cross-sectional household survey named the Multiple Indicator Cluster Survey (MICS) Bangladesh 2019. A total of 58,746 children aged 5-17 were selected for the study, where 30,300 children were male, and 28,446 were female. To deal with overdispersed count data, the study used a negative binomial regression model to find the associated factors. The results show that 39.3% of the male children and 40.9% of the female children were from the age group of 10-14. Educated children had a lower risk of dysfunction. Among male children, women with a total number of children ever born of 4 or more were 1.21 times (incidence rate ratios (IRR) = 1.21) more likely to have a dysfunctional child. Children of dysfunctional mothers are more likely to be dysfunctional themselves. The incidence rate ratio for children functional difficulty among Muslim girls was 36 percent higher than non-Muslim girls in Bangladesh. When compared to the Barisal Division, female children in the Mymensingh Division had a 16% higher risk of functional problems. Based on the findings, the Bangladesh government and other development partners should initiate policies and programs to minimize the impact of functional dysfunction in children.

## 1. Introduction

Around the world, significant progress in maternal and child well-being has been made over the past two decades [1, 2]. Despite these significant advances in health and well-being, there is not much progress in functional difficulties in children. When a person may have activity limitations, then this limitation is called functional difficulties [3]. A person has experienced limitations due to their medical condition, such as severe or moderate illness, short- or long-term injuries, mental or emotional issues, etc.

Currently, the functional difficulty of the child is a hot topic. It is also associated with psychological morbidity [4]. Children with functional difficulties or disabilities have a higher risk of psychological morbidity [5]. Mentally healthy children enjoy a good quality of life and can function well at home, in school, and communities [6]. For children who have functional difficulties, their overall improvement is less than others. A study revealed that child activity and well-being impacted child development [7]. A recent study by Hossain et al. [8] showed that the child development score is not satisfactory in Bangladesh. The functional difficulty is one of the reasons for this unsatisfactory finding. Another study in Bangladesh found that children with functional difficulties had 76 percent less development over 36-59 months [9]. So, it is a well-known fact that the functional difficulties of the child are an important indicator of children's future development.

Functional difficulties are major health problem for children around the world, particularly in low- and middle-income countries. Globally, 15% of people have functional difficulties, and 150 million children live with a disability [10]. In the United States, around 35% of youth with disabilities had a learning disability, 21% had a speech or language impairment, 7% had an intellectual disability, and around 5% had an emotional disability [11].

In sub-Saharan Africa, 66 percent of the children are at the risk of not reaching their developmental potential, and only 57 percent of children in low-income and middle-income countries fulfilled their development potential [12]. Although much research work has done on child development in Bangladesh, very little has been done on the functional difficulties of children. Past investigation using multiple indicator cluster survey also mentioned that child functional difficulties are a global health concern. According to a Bangladesh MICS survey, around 3% of children aged 2-4 have severe functional difficulties, while 8% of children aged 5-17 [13].

Children's functional difficulties and disabilities are a global burden, and children's health is one of the most important issues in the sustainable development goals (SDGs) [14]. Dysfunction substantially affected the developmental phase of the children, as the children grow up with dysfunction experience, worse socioeconomic outcomes, and deprived from basic rights. There are various factors that influence the functional difficulties of children. Previous studies identified that child-level factors (child age, child sex), household-level factors (wealth status), maternal and paternal level factors (maternal education, paternal education, maternal health status, and so on), and community-level factors (place of residence, regional variations) are the important factors of child functional difficulties [15–19].

Early identification of children at risk of dysfunction allows for intervention and remediation, thereby potentially reducing adverse effects on educational attainment and future well-being. In the past, several researchers have tried to determine the actual risk factors for child disabilities by using various techniques and statistical models. A recent study conducted by Dey et al. in Ghana used the Poisson regression model [20]. But based on the dispersion test suggested by Cameron and Trivedi [21], the current study used the negative binomial regression model to fit overdispersed data. The main purpose of this study is to find out the prevalence of children's functional difficulties and their associated factors in Bangladesh. The study will play an important role in setting national priorities in the policies for the development of children with dysfunction and in formulating, implementing, and evaluating sustainable development plans.

## 2. Materials and Method

### 2.1. Data Source

**Figure 1:**
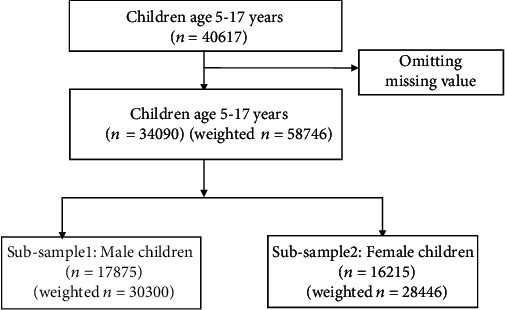
Workflow of diagram of the study.

This study used data from the Multiple Indicator Cluster Survey (MICS) from Bangladesh in 2019. MICS is a nationally representative cross-sectional household survey program initiated in the 1990s by the United Nations International Children's Fund (UNICEF) to collect data on key indicators of the well-being of children and women in several countries. Bangladesh MICS 2019, conducted by the Bangladesh Bureau of Statistics (BBS) in collaboration with UNICEF Bangladesh, uses a two-stage stratified cluster sampling procedure to collect data. The rural and urban areas within each district of Bangladesh were identified as the main sampling strata. In the first stage, a specified number of census enumeration areas (EA) were selected systematically with probability proportional to size within each stratum, known as the primary sampling unit (PSU). A household listing was carried out within each EA to provide a sampling frame for the second stage. The systematic sampling was then used to select 20 households from each PSU sample. Thus, Bangladesh MICS 2019 covered 64,400 residential households. Data are obtained from 40,617 children between the ages of 5 and17 years. Data weighted for this analysis according to the MICS guidelines, and therefore the final sample size for this study was 58,746 children aged 5-17, of whom 30,300 were males, and 28,446 were females. Following Figure 1 shows the flowchart of the study.

### 2.2. Study Variable

#### 2.2.1. Dependent Variable

The dependent variable for the study was the functional difficulty status of children aged 5-17 years. The Bangladesh Multiple Indicator Cluster Survey 2019 included child functioning modules to assess child functional difficulties as a child experiencing any impairment in the following 13 domains: seeing, hearing, walking, self-care, communication, learning, remembering, concentrating, accepting change, controlling behavior, making friends, anxiety, and depression. The responses of “A lot of difficulties,” “Cannot at all,” or “Daily” and “Weekly” to the above domains were considered impairments of the child and recoded as 1 for “Yes”, otherwise 0 for “No”. Finally, the functional difficulty variable was measured as a count variable, which counts the total number of difficulties out of these 13 domains as 0, 1, 2, ⋯, 13.

#### 2.2.2. Independent Variable

Independent variables were child age (5-9 years, 10-14 years, and 15-17 years), division (Barishal, Chattogram, Dhaka, Khulna, Mymensingh, Rajshahi, Rangpur, and Sylhet), mother's disability (Yes and No), place of residence (urban, rural), child education (no education, ECE, primary, secondary, and secondary+), religion (Muslim and others), household head education level (preprimary or none, primary, and secondary+), child ever born (1, 2-3, and 4+), wealth index (poor, middle, rich), and mother's age (15-24 years, 25-34 years, and 35+ years).

### 2.3. Statistical Analysis

Gender inequality, discrimination against girls, and son preference still exist in most countries, which affect the care for girls' right from birth. Thus, for the study, analysis was divided into two sections, one for male children and the other one for female children to identify the risk factors associated with children's functional difficulties separately. For the study variable, functional difficulty is a count variable; the following statistical models were used:

1. The Poisson regression model
2. Negative binomial regression model

#### 2.3.1. The Poisson Regression Model

The Poisson regression model is the widely used model for dealing count data [22]. The model is also called a log-linear model, as the logarithm of the conditional mean is a linear function of the predictors [21]. Let *Y*_1_, *Y*_2_, ⋯, *Y*_*n*_ be the *n* independent random variables where *y*_*i*_ denotes the total functional disabilities of *i*^th^ child. Let *X*_*i*_ denote a vector of explanatory variables.

Then, the Poisson equation of *Y*_*i*_ with rate parameter *λ*_*i*_ is given by

(1) $$\begin{array}{c} P\left[Y_{i}=y_{i}\right]=\frac{e^{-\lambda_{i}}\lambda_{i}^{y_{i}}}{y_{i}!}. \end{array}$$

Here, *λ*_*i*_ > 0 and *y*_*i*_ = 0, 1, 2, ⋯, ∞.

The mean parameter

(2) $$\begin{array}{c} E\left[\left.y_{i}\right|x_{i}\right]=\lambda_{i}=\exp\left(x_{i}^{\prime }\beta \right). \end{array}$$

#### 2.3.2. Negative Binomial Regression Model

Since the standard Poisson regression model has the assumption of equidispersion thus, the effect of overdispersion can lead to inefficient estimation made under the Poisson model. Negative binomial regression model is perhaps the most convenient to deal with overdispersed count data. The negative binomial has an additional heterogeneity parameter that accommodates dispersion in the data [23].

Let *Y*_1_, *Y*_2_, ⋯, *Y*_*n*_ be a set of *n* independent random variables, then the negative binomial model given as

(3) $$\begin{array}{c} P\left[Y_{i}=y_{i}\right]=\frac{\Gamma \left(y_{i}+\left(1/\alpha \right)\right)\;}{\Gamma \left(y_{i}+1\right)\;\Gamma \left(1/\alpha \right)}\;{\left(\frac{\alpha \lambda_{i}}{1+\alpha \lambda_{i}}\right)}^{y_{i}}\;{\left(\frac{1}{1+\alpha \lambda_{i}}\right)}^{1/\alpha }, \end{array}$$

where *α* ≥ 0 is often referred to as the index or dispersion parameter, and *y*_*i*_ = 0, 1, 2, ⋯⋯ The mean and variance of *Y*  are

(4) $$\begin{array}{c} E\left(\left.Y\right|x\right)=\lambda \text{ and Var}\left(\left.Y\right|x\right)=\lambda +\alpha \lambda^{2}. \end{array}$$

#### 2.3.3. Test for Overdispersion

When the dispersion parameter *α* = 0, the Negative binomial model reduces to the Poisson model. Thus, it is necessary to check the dispersion test statistics whether there is any over dispersion or under dispersion in the data. A dispersion test is performed to assess the assumption of equidispersion of the Poisson model [21]. A null hypothesis *H*_0_ : *α* = 0  can be set to measure the presence of dispersion. The rejection of the null hypothesis indicates that the data is dispersed. Therefore, the presence of the dispersion parameter *α* in the negative model is justified.

#### 2.3.4. Model Performance Evaluation

Model performance evaluation was used to select the best statistical model that fit the data well. To assess the performance of the Poisson regression model and the negative binomial regression model, zero-inflation ratio was used to check whether the count models are over or under fitting zeros in the outcome. A ratio of observed zeros and predicted zeros between 1 + /−tolerance is regarded as acceptable, while a ratio beyond or below this threshold would indicate over or underfitting. Usually, the tolerance is set to 0.05. Furthermore, the Akaike information criterion (AIC) and Bayesian information criterion (BIC) were also used to identify the goodness of fit of the model.

### 2.4. Analytical Software

For data management and analysis, SPSS software (IBM Corporation, Armonk, New York, NY, USA) version 20.0 and R programming version 4.0.0 were used.

## 3. Results

### 3.1. Demographic and Socioeconomic Characteristics

Table 1 shows the frequency of different selected indicators for functional difficulties of male and female children aged 5-17 in Bangladesh. A total of 58,746 children aged 5-17 were included in the study. Among them, 30,300 children were male, and 28,446 children were female. Only 19.8% of the children were in the age group of 15-17. Half of the male children (50.5%) completed primary level education, whereas 46.4% of the female children completed so. The percentage of un-educated male children (5.3%) was slightly higher than that of female children (3.9%). More than half of the children's mothers (51.1%) were aged between 35 and 49 years. More than three-fifths of the children's mothers (61.7%) had parity 2-3. Almost all the mothers of the children (96.7%) did not have functional difficulties. Most of the children (79.2%) were from rural areas, and most (91.2%) were Muslims. 42.6% of the children were from poor households, while 37.8% were from rich households.

### 3.2. Test for Overdispersion and Model Selection

Table 2 shows a significant dispersion test, which indicates that the overdispersion was present in the data set. The zero-inflation ratio of the negative binomial regression model illustrates that the ratio of observed and predicted zero is almost the same, which is considered a good fit. Furthermore, the negative binomial regression model had the smallest AIC and BIC.

### 3.3. Identify the Contributing Factors to Child Functional Difficulty

The estimated rate ratio for the analysis of functional difficulties in children in sociodemographic factors with gender differences in Bangladesh using the negative binomial regression model (with 95% CI and *p*-value) is presented in Table 3. The factors that were significantly associated with the study outcome were child age, child education, maternal age, child ever born, maternal disability, educational status of household head, residence, religion, and division.

Study found that children aged 15 to 17 years were at increased risk of dysfunction in male and female children compared to children under 10 years of age. More specifically, compared to children under 10, children over 15 years of age had a 41 percent (male) and 77 percent (female) more risk of dysfunction. Educated children had a lower risk of dysfunction compared to children without education. Male children from adult mothers (25-34 and 35+ age) had a lower risk of dysfunction than that young mother (15-24 years of age). On the other hand, female children of mother aged 25 to 34 had a 25 percent higher risk of dysfunction as compared to young mother. Among male children, whose mother had 4+ ever born child were 1.21 times (IRR = 1.21) more likely to had a child dysfunction compared to children of primiparous mother. The negative binomial regression model's result shows that children of dysfunctional mothers are more likely to had dysfunction.

The level of education of the household head is an important indicator related to the dysfunction of children, especially boys. Children from households whose head had no education and primary education were at higher risk of having dysfunction than children of household heads with secondary or above education. The results of this study show that Muslim boys had a higher risk of dysfunction than non-Muslim boys by 15%. On the other hand, this incidence rate ratio for Muslim girls was significantly 36% higher than the non-Muslim girls in Bangladesh.

The results obtained from the male children show that the children living in Chattogram, Dhaka, Khulna, Mymensingh, Rangpur, Rajshahi, and Sylhet had significantly fewer dysfunction compared to the children living in the Barisal Division. For example, those living in the Rajshahi division had a 51% lower risk of child functional difficulty than those living in the Barisal Division. Compared to the Barishal division, boys from Sylhet (91%), Rangpur (86%), Dhaka (81%), and Khulna (80%) had the lowest risk of functional problems, whereas female children from Mymensingh division had a 16% higher risk of functional difficulty than children who reside in Barishal division. In addition, this risk was lower in other divisions (such as Chattogram, Dhaka, Khulna, Rajshahi, Rangpur, and Sylhet) compared with the Barishal division. Again, child functional difficulties were higher in rural areas, especially for girls. The risk faced by girls in rural areas was significantly higher (IRR = 1.12) than in urban areas.

## 4. Discussion

This is one of the few studies carried out among Bangladeshi children using negative binomial regression to assess the extent and factors associated with children's functional difficulties, both in male and female children of Bangladesh. This study was carried out among 58,746 children aged 5-17, where 30,300 children were male, and 28,446 were female from a nationally representative survey named Bangladesh Multiple Indicator Cluster Survey, 2019.

This article focused on identifying risk factors associated with functional difficulties in children for both male and female children in Bangladesh. Children's functional difficulties are a burning issue in our country. A study revealed that normal children were less likely to be abused or ignored than children with disabilities [24, 25]. Additionally, misconduct changes according to the type of crippled child among disabled children [25–27].

Several factors were identified that were associated with children's disabilities in this analysis. Child functional difficulties status was significantly related to child age, child education, mother age, mother disability, religion, and division for both male and female children. Children ever born, household head education level, and functional disability of mothers had a significant impact on children's functional disability of children for male children, while only place of residence had a significant impact on children's functional disability of children for female children.

Child age is significantly positively associated with children's functional difficulties in both female and male children in Bangladesh. In this analysis, the older children were more likely to have functional difficulty compared to the younger children in this analysis. Children aged 10 years or younger or those with other causes of ID were used twice as much with lower service complexity as children aged 11-14 years and those with autism spectrum disorder [28]. However, a previous study conducted in Ghana does not support our findings and found no significant association between child age and functional difficulties in children [20].

In terms of child education, children without education were more likely to suffer functional difficulties compared to educated children of both sexes. In a different context, evidence from child nutrition status researchers observed that children who had an education were less likely to be malnourished compared to uneducated children, for both male and female children [29]. Disabled children also have poor health and educational outcomes [30]. On the other hand, there was no significant association between child education and children's functional difficulty [20].

Increased mother age can decrease the likelihood that children have functional difficulty in male children. However, it is the opposite for female children. Furthermore, children of mothers with 4 or more children were more likely to suffer from functional difficulties compared to their reference category. A previous study from the United States also found that parental mental health, the number of children, and family structure had consistent correlations with child health outcomes [31, 32].

In our analysis, the mother's disability is a significant risk factor for the condition of functional difficulty in children. This evidence coincides with other studies conducted in Ghana and found that when a mother has a disability, a child is more likely to develop a disability than a child who does not have a disabled mother [20]. Evidence from earlier literature suggests that parents of disabled children, particularly mothers, are more likely to suffer from mental health problems [33, 34] and physical health problems [35].

Study also found that household head education is a potential risk factor related to the dysfunction of children, especially boys. This finding was not supported by previous research conducted by Rai et al., [36] in Sweden and revealed that there were no significant links found between parental education and offspring autism spectrum disorders [36]. According to health care professionals, children from educated families were more likely to have had respiratory, food, or skin allergies in the past 12 months [32]. This is a very interesting finding and similar to our study, which found that higher levels of parental education have consistently been linked to better child development outcomes [37].

The results of this study show that Muslim children have a higher risk of dysfunction than non-Muslim children. Rural women were more likely to have functional difficulties compared to urban women. However, in the study of Bangladeshi children's nutrition status, researchers found similar results, observing that female children in rural areas were more likely to be malnourished compared to urban children [29, 38].

In this analysis, the division in which a child lives had a significant relationship with the functional difficulty of the child. Findings demonstrate that all of the divisions except the Mymensingh division were significantly associated with children's functional difficulties in Bangladesh. The risk of having male children with functional difficulties was relatively lower in all divisions except for the Sylhet division compared to female children in this analysis. However, a study conducted in Bangladesh showed that children in the Dhaka division were less likely to be malnourished than children in the Sylhet division in Bangladesh [39]. A previous study conducted in Ghana showed that the region is a significant risk factor for children's functional difficulties [20].

## 5. Conclusion

The present study examines the factors associated with functional difficulty for both male and female children aged 5 to 17 years. Children of disable mothers, rural children, Muslim children, and children of a mother having an order of parity of 4 or more were at higher risk of dysfunction. Moreover, female children of aged mothers and male children of the young mother were at higher risk of dysfunction. On the contrary, higher education for children and the head of the household lowers the likelihood of dysfunction. To address dysfunction problems, a platform should be formed in Bangladesh with collaborative efforts and resource generation between government and nongovernmental sectors. Furthermore, child welfare professionals can play a significant role in developing supportive relationships for disabled children and their families by identifying and addressing family strengths and needs, so that a child with functional difficulties can live in safe and supportive environments.

## Figures and Tables

**Table 1:** Demographic and socioeconomic characteristics of the selected covariates.

Variable	Male (*n* = 30,300)	Female (*n* = 28,446)	Total (*n* = 58,746)
	Frequency (%)	Frequency (%)	Frequency (%)
Child age (in years)			
5-9	11831 (39.1)	11791 (41.5)	23622 (40.2)
10-14	11895 (39.3)	11626 (40.9)	23521 (40)
15-17	6574 (21.7)	5029 (17.7)	11603 (19.8)
Child education			
No education	1598 (5.3)	1116 (3.9)	2714 (4.6)
ECE	2768 (9.1)	2522 (8.9)	5290 (9.0)
Primary	15312 (50.5)	13202 (46.4)	28514 (48.5)
Secondary	6334 (20.9)	6918 (24.3)	13252 (22.6)
Secondary+	4287 (14.2)	4688 (16.5)	8975 (15.3)
Mother age (in years)			
15-24	1252 (4.1)	1184 (4.2)	2436 (4.1)
25-34	13521 (44.6)	12798 (44.9)	26319 (44.8)
35+	15527 (51.2)	14464 (50.9)	29991 (51.1)
Child ever born			
1	2326 (7.7)	1838 (6.5)	4164 (7.1)
2-3	19165 (63.2)	17107 (60.1)	36272 (61.7)
4+	8808 (29.1)	9502 (33.4)	18310 (31.2)
Mother disability			
Yes	1020 (3.4)	944 (3.3)	1964 (3.3)
No	29279 (96.6)	27502 (96.7)	56781 (96.7)
HH education level			
Preprimary or none	10744 (35.5)	9667 (33.9)	20411 (34.7)
Primary	8571 (28.3)	8066 (28.4)	16637 (28.3)
Secondary+	10984 (36.2)	10714 (37.7)	21698 (36.9)
Religion			
Muslim	27662 (91.3)	25928 (91.1)	53590 (91.2)
Other	2638 (8.7)	2518 (8.9)	5156 (8.8)
Place of residence			
Urban	6384 (21.1)	5828 (20.5)	12212 (20.8)
Rural	23915 (78.9)	22618 (79.5)	46533 (79.2)
Wealth index			
Poor	13052 (43.1)	11963 (42.1)	25015 (42.6)
Middle	6112 (20.2)	5396 (18.9)	11508 (19.6)
Rich	11136 (36.7)	11087 (38.9)	22223 (37.8)
Division			
Barishal	1633 (5.4)	1671 (5.9)	3304 (5.6)
Chattogram	6460 (21.3)	6343 (22.3)	12803 (21.8)
Dhaka	7196 (23.8)	6712 (23.6)	13908 (23.7)
Khulna	3149 (10.4)	2942 (10.3)	6091 (10.4)
Mymensingh	2124 (7)	2038 (7.2)	4162 (7.1)
Rajshahi	3671 (12.1)	3310 (11.6)	6981 (11.9)
Rangpur	3452 (11.4)	3043 (10.7)	6495 (11.1)
Sylhet	2615 (8.6)	2387 (8.4)	5002 (8.5)

HH = household head; ECE = early child education.

**Table 2:** Model selection criterion for regression models.

Criterion	Poisson regression model		Negative binomial regression model	
	Male	Female	Male	Female
Dispersion test	*p* value < 0.001	*p* value < 0.001	—	—
Zero inflation ratio	0.90	0.91	0.99	0.99
AIC	40940	36800	33042	30512
BIC	41135	36993	33244	30712

**Table 3:** Negative binomial regression model for children aged 5 to 17 years.

Predictors	Male		Female	
	IRR [95% CI]	*p* value	IRR [95% CI]	*p* value
Child age (in years)				
5-9 (ref.)	1.00		1.00	
10-14	1.27 [1.15–1.41	<0.001	1.39 [1.25–1.55	<0.001
15-17	1.41 [1.23–1.61	<0.001	1.77 [1.50–2.09	<0.001
Child education				
No education (ref.)	1.00		1.00	
ECE	0.2 [0.17–0.24]	<0.001	0.23 0.19–0.28]	<0.001
Primary	0.2 [0.17–0.23]	<0.001	0.15 [0.13–0.18]	<0.001
Secondary	0.15 [0.13–0.17]	<0.001	0.1 [0.09–0.12]	<0.001
Secondary+	0.12 [0.10–0.14]	<0.001	0.1 [0.08–0.13]	<0.001
Mother age (in years)				
15-24 (ref.)	1.00		1.00	
25-34	0.74 [0.61–0.88]	0.001	1.25 [1.03–1.52]	0.027
35+	0.62 [0.51–0.75]	<0.001	1.1 [0.89–1.35]	0.388
Child ever born				
1 (ref.)	1.00		1.00	
2-3	1.07 [0.93–1.24]	0.348	1.12 [0.95–1.31]	0.18
4+	1.21 [1.03–1.43]	0.023	1.16 [0.97–1.38]	0.1
Mother disability				
Yes (ref.)	1.00		1.00	
No	0.38 [0.32–0.45]	<0.001	0.37 [0.31–0.43]	<0.001
HH education level				
Preprimary or none	1.12 [1.02–1.23]	0.023	1.04 [0.94–1.14]	0.463
Primary	1.1 [1.00–1.21]	0.049	1.01 [0.92–1.11]	0.795
Secondary+ (ref.)	1.00		1.00	
Religion				
Muslim	1.15 [1.00–1.33]	0.046	1.36 [1.17–1.57]	<0.001
Other (ref.)	1.00		1.00	
Place of residence				
Urban (ref.)	1.00		1.00	
Rural	1.09 [0.98–1.20]	0.101	1.12 [1.01–1.24]	0.029
Wealth index				
Poor	1.19 [0.83–1.02]	0.121	1.1 [0.96–1.18]	0.213
Middle	1.08 [0.90–1.11]	0.996	1.06 [0.89–1.10]	0.847
Rich (ref.)	1.00		1.00	
Division				
Barishal (ref.)	1.00		1.00	
Chattogram	0.37 [0.32–0.43]	<0.001	0.46 [0.40–0.53]	<0.001
Dhaka	0.19 [0.17–0.23]	<0.001	0.24 [0.21–0.28]	<0.001
Khulna	0.2 [0.16–0.23]	<0.001	0.23 [0.20–0.28]	<0.001
Mymensingh	0.86 [0.73–1.02]	0.043	1.16 [0.99–1.36]	0.038
Rajshahi	0.49 [0.42–0.57]	<0.001	0.51 [0.44–0.60]	<0.001
Rangpur	0.14 [0.11–0.16]	<0.001	0.19 [0.16–0.23]	<0.001
Sylhet	0.09 [0.07–0.11]	<0.001	0.08 [0.06–0.10]	<0.001

ref.=reference category; HH = household head; ECE = early child education; IRR = incidence rate ratios.

## Data Availability

Data associated with this study is available at https://mics.unicef.org/.
